# The work and training situation for young physicians undergoing specialty training in gynecology and obstetrics in Germany: an assessment of the status quo

**DOI:** 10.1007/s00404-020-05616-0

**Published:** 2020-05-26

**Authors:** Johannes Lermann, Julia Knabl, Johannes Neimann, Kevin Schulte, Kim Proske, Sarah Schott, Matthias Raspe

**Affiliations:** 1 Department of Gynecology and Obstetrics, Erlangen University Hospital, Friedrich Alexander University of Erlangen–Nuremberg, Universitätsstrasse 21-23, 91054 Erlangen,, Germany; 2 Department of Obstetrics and Perinatal Medicine, Hallerwiese Clinic, Nuremberg, Germany; 3 Specialist in Gynaecology and Obstetrics, 31228 Peine, Germany; 4 Department of Internal Medicine IV/Renal and Hypertensive Diseases, Schleswig-Holstein University Hospital, Kiel Campus, Kiel, Germany; 5 Department of Gynecology and Obstetrics, Heidelberg University Hospital, Heidelberg, Germany; 6 Department of Infectious Diseases and Respiratory Medicine, Charité-Universitätsmedizin Berlin, Corporate Member of Freie Universität Berlin, Humboldt-Universität zu Berlin, and Berlin Institute of Health, Charitéplatz 1, 10117 Berlin, Germany

**Keywords:** Gynecology and obstetrics, Medical specialty training, Working conditions, Stress factors, Psychosocial work pressure

## Abstract

**Purpose:**

General conditions in the health-care system in Germany have changed dramatically in recent years. Factors affecting this include above all demographic change, rapid developments in diagnostic and therapeutic options, and the application of economic criteria to the health-care sector. This study aimed to establish the current status quo regarding conditions of work and training for young doctors in gynecology and obstetrics, analyze stress factors, and suggest potential improvements.

**Methods:**

Between October 2015 and March 2016, a web-based survey was carried out among residents and members of the German Society for Gynecology and Obstetrics. The electronic questionnaire comprised 65 items on seven topics. Part of the survey included the short version of a validated model of professional gratification crises for analyzing psychosocial work-related stress.

**Results:**

The analysis included a total of 391 complete datasets. Considerable negative findings in relation to psychosocial work pressure, time and organizational factors, quality of specialty training, and compatibility between work and family life and work and academic tasks were detected. A high level of psychosocial work pressure is associated with more frequent job changes, reduced working hours, poorer health among physicians, and a lower subjectively assessed quality of care.

**Conclusions:**

Greater efforts are needed from all the participants involved in patient care to achieve high-quality training and working conditions that allow physicians to work in a healthy and effective way. These aspects are all prerequisites for sustainably maximizing the resource “physician” and for ensuring high-quality patient care.

## Introduction

General conditions in the health-care system in Germany have changed dramatically in recent years. Demographic changes, increasing subspecialization, and above all the implementation of the German diagnosis-related groups (G-DRG) system—with the resulting intensification of expected performance levels and commercialization—have ultimately led to the focus of work being diverted away from the patient and toward goals such as increasing productivity and profits, and reducing costs [1–3]. For young physicians working in hospitals in Germany, these developments are above all perceived as involving a severe intensification of the workload [4, 5].

This intensification of work, combined with a heavy burden in terms of time and psychosocial work pressure, as well as steep organizational hierarchies, can cause physicians to become frustrated and move away from direct patient care [6]. The shortage of adequate medical working time has already become evident as a result of errors in care [7]. Consistently with developing staff shortages, the numbers of foreign physicians working in Germany increased from 10,989 in 1998 (corresponding to 3.8% of all doctors in Germany) to 48,672 in 2018 (12.4% of all physicians) [8]. Currently, 82% of specialists up to and including age 39 working in the field of gynecology and obstetrics are female [8]. The major reasons for physicians’ choice of hospitals today—in addition to the usual criteria such as salary levels, location, and hospital size—are the availability of clearly structured medical specialty training curricula with predetermined goals and agreements, opportunities for promotion and development, and career options that also include part-time employment [9]. This places pressure on employers in the health-care system to respond receptively to the changing demands of the younger generation of doctors.

Gynecology is facing special challenges in this context, since in comparison with other medical disciplines particularly large numbers of female physicians opt for this specialization, and in addition it involves a high proportion of night work and shift work (especially in obstetrics). The high proportion of female physicians in gynecology and obstetrics, along with changing role models, is leading to high levels of part-time employment—primarily among female doctors, but increasingly also among male doctors. In combination with night work and shift work, this is creating additional challenges in relation to organizing the workplace in conformity with legal obligations and fulfilling the catalog of surgical procedures required for specialist medical qualifications [10]. For the latter, a large number of examination techniques and interventions have to be learned and reliably mastered within a planned training period of 60 months. The current amendment of the (model) statutory professional medical education regulations is intended to adapt the numbers required to the reality in hospitals in Germany—but what is the reality?

There is a need for up-to-date surveys to be carried out to provide a basis for current discussions on working conditions and further training conditions, and to make it possible to develop strategies for improving the field of gynecology and obstetrics. The survey presented in this article was conducted to analyze the current situation in the key areas of working life for clinical doctors in gynecology and obstetrics, to identify factors particularly requiring improvement, and to deduce the appropriate steps needed to correct existing deficiencies.

## Methods

### Sample and implementation

All physicians registered with the German Society for Gynecology and Obstetrics (*Deutsche Gesellschaft für Gynäkologie und Geburtshilfe e.V., *DGGG) who were currently in residency were invited via e-mail by the society’s main office to take part in an anonymized survey, followed by two reminder e-mails. Participation was possible between October 1, 2015 and March 31, 2016 (6 months). The opportunity to participate was also advertised in the printed journal *Frauenarzt*, via e-mail distribution lists, and on the home page of the DGGG’s “Young Forum,” as well as at conferences. The survey was conducted through an online survey provider, SurveyMonkey^®^ (Survey Monkey Inc., San Mateo, CA, USA). Each study participant agreed to data analysis prior to starting the study.

### Questionnaire

The questionnaire included 65 items on seven subject areas. These were, in detail: (1) working conditions in everyday professional life: 11 questions; (2) continuing professional education and specialty training: 11 questions; (3) operations and procedures in gynecology and obstetrics: 12 questions; (4) compatibility of work and family life: nine questions; (5) compatibility of work and research: six questions; (6) model for professional gratification crises: 16 questions; and (7) basic information: nine questions.

The questionnaire was partly based on a survey conducted among internal medicine residences in 2014 [11]. The short version of the validated effort–reward imbalance (ERI) questionnaire, also known as the Model of Professional Gratification Crises, including 16 items, was incorporated into the survey, with subscales for effort (three items), reward (seven items), and overcommitment (six items), on a four-point Likert scale [12–14]. The values for questionnaire version and scaling were made comparable nationally and internationally through adjustment to values between 0 and 100. On the basis of the adjusted scores from the effort and reward subscales, a quotient following the model was created to allow quantitative estimation of the extent of a professional gratification crisis (the gratification crisis quotient, or effort–reward ratio). A value greater than 1 indicates a preponderance of effort factors and thus a gratification crisis or its synonym, a high level of psychosocial work pressure.

### Statistics

As parametric methods of statistical hypothesis testing, the *t* test for independent samples (with 95% confidence intervals) was used for comparison of two groups and analysis of variance (ANOVA) with the Tukey post-hoc test was used for more than two groups. Nonparametric tests used were the Mann–Whitney *U* test (MWU) and the Kruskal–Wallis test (with MWU tests for post-hoc analysis). Expected and observed distribution patterns were compared using cross-tabulation and checked for statistical significance using the Chi-squared test. Pearson’s *r* or Spearman’s rho were used for correlations. A *P* value ≤ 0.05 was considered statistically significant. The following measures of effect size were used for the above tests: Cohen’s *d* for *t* test (0.5 smaller, 0.5–0.8 moderate, > 0.8 strong effect), eta-squared (*η*^2^) for ANOVA (< 0.06 smaller, 0.06–0.14 moderate, > 0.14 strong effect), *r* for MWU test (< 0.3 smaller, 0.3–0.5 moderate, > 0.5 strong effect), Cramér’s *V* for Chi-squared (0.1 small, 0.3 moderate, 0.5 strong effect) and Pearson’s *r* and Spearman’s rho for correlations (< 0.3 small, 0.5 moderate, > 0.7 high correlation). Adjustment for multiple testing was carried out using the Bonferroni–Holm method (starting from significance level *α* = 0.05, 22 statistical hypothesis tests over the whole sample, new significance level indicated as *α*_*x*_, where appropriate). In all of the tests applied, the parametric and nonparametric methods gave a consistent result (e.g. *t* test vs. MWU test, ANOVA vs. Kruskal–Wallis test). For reasons of clarity, only the test method applied primarily is specified in the Results section. All of the statistical analyses were carried out using IBM SPSS Statistics for Windows, version 25 (IBM Corporation, Armonk, NY, USA).

## Results

### Response and basic data

A total of 585 physicians receiving medical specialty training started the survey, and 391 participants fully completed it. Among the other participants, 65% (126/194) abandoned the questionnaire before the seventh question. The basic data (*n* = 391) from the present survey are shown in Table 1.

**Table 1:** Basic data for the 391 participants in the survey

Total participants	391
Gender (F/M, %)	86/14
Age in years (mean ± SD)	31.5 ± 3.8
Year of specialty training (mean ± SD)	3.6 ± 1.8
Working hours	
Full-time/part-time (%)	70/30
Full-time, female: male (%)	66:96
Part-time, female: male (%)	34:4
Child(ren) (no/yes, %)	62/38
Nationality (German/other, %)	92/8
Federal state (%, three most common)	
Bavaria	26
North Rhine–Westphalia	17
Baden-Wurttemberg	17
Main field of work (%, three most common)	
General gynecology	35
Obstetrics	34
Gynecological oncology	16
Hospital ownership structure (%)	
Public	57
Charitable	30
Private	13
Hospital level of care	
Basic care	43
Maximum care	30
University	24

### Model of professional gratification crises

Table 2 provides an overview of the 16 questions in the short version of the model of professional gratification crises, with the corresponding distribution of responses. The adjusted values for the subscales (i.e., adjusted for the scope of the questionnaire version and scaling so as to be comparable with other national and international studies with scores between 0 and 100), and the gratification crises quotient (synonym: ER ratio) derived from them, were calculated as a measure of psychosocial work pressure. Psychosocial work pressure was high, with an ER ratio of 1.8 ± 1.1 (mean ± standard deviation; effort scale 79 ± 17, reward scale 51 ± 16). Eighty-two percent of the respondents had an ER ratio > 1, and 30% had an ER ratio > 2. The more advanced the participants were in their medical specialty training (divided into three categories, first to third years, versus fourth to fifth years, versus sixth or later year of specialty training), the greater was the level of psychosocial work pressure (ER ratio relative to professional training period: 1.62 vs. 1.97 vs. 2.12, ANOVA/Tukey, *P* = 0.003, *α*_7_ 0.007, eta-squared 0.03; fourth to fifth vs. first to third years, *P* = 0.012; 95% CI, 0.06–0.63; ≥ sixth vs. first to third years, *P* = 0.024; 95% CI 0.05–0.95; ≥ sixth vs. fourth to fifth years, *P* = 0.71). The reward scale can be differentiated into the three factors esteem, promotion, and security. Comparing these factors resulted in lower values for esteem (two items, 4.6 ± 1.5, range 2–8; mean/maximum score: 58%) and promotion opportunities (three items, 7.3 ± 1.6, range 3–12, 61%) than for security (two items, mean 5.9 ± 1.2, range 2–8 points, 74%). The overcommitment value (overall 54 ± 17) was also prominent, without gender differences (*t* test, *P* = 0.98).

**Table 2:**
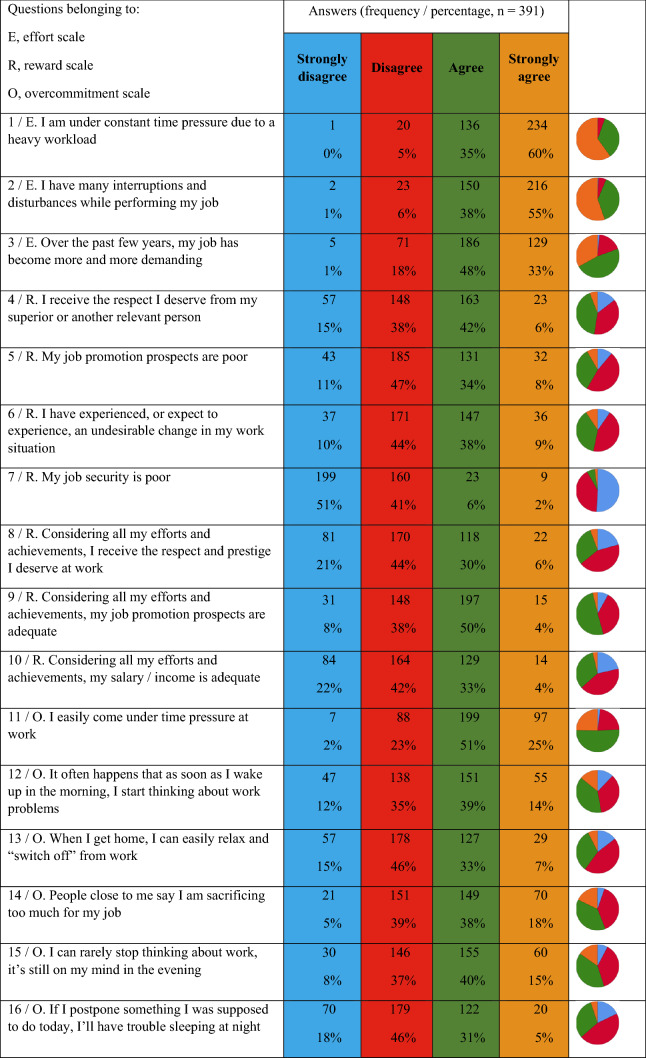
Responses of the 391 participants to the 16 questions in the short version of the model of professional gratification crises

### Conditions in everyday working life

Asked about satisfaction with their current job situation, 6% (22/391) stated they were very satisfied, 34% (134/391) fairly satisfied, 34% (132/391) undecided, 20% (79/391) fairly dissatisfied, and 6% (24/391) very dissatisfied. The three most common reasons given for dissatisfaction (only undecided and dissatisfied included, 14 reasons to choose, a maximum of three choices, on average 2.9 responses per participants) were the poor quality of specialty medical education and irregular working hours, both at 13%, and long working hours, at 12%. The more advanced the respondents were in their specialty training, the lower their level of job satisfaction (Kruskal–Wallis/MWU, *P* < 0.001, with further training time divided into the three sections first to third, fourth to fifth, and sixth or later years of further education).

The participants were asked to estimate the proportion of their daily working hours relative to proximity to the patient. The proportion of daily working hours spent with the patient or at the patient’s bedside was 44% ± 16% (e.g., for surgery, doctor’s visits, physical examinations). The proportion of patient-related work (e.g., writing medical letters, taking part in case review discussions) was 35% ± 12%, and the proportion of nonmedical activities in the strict sense (e.g., coding, organizing examinations) was 22 ± 10%.

**Fig. 1:**
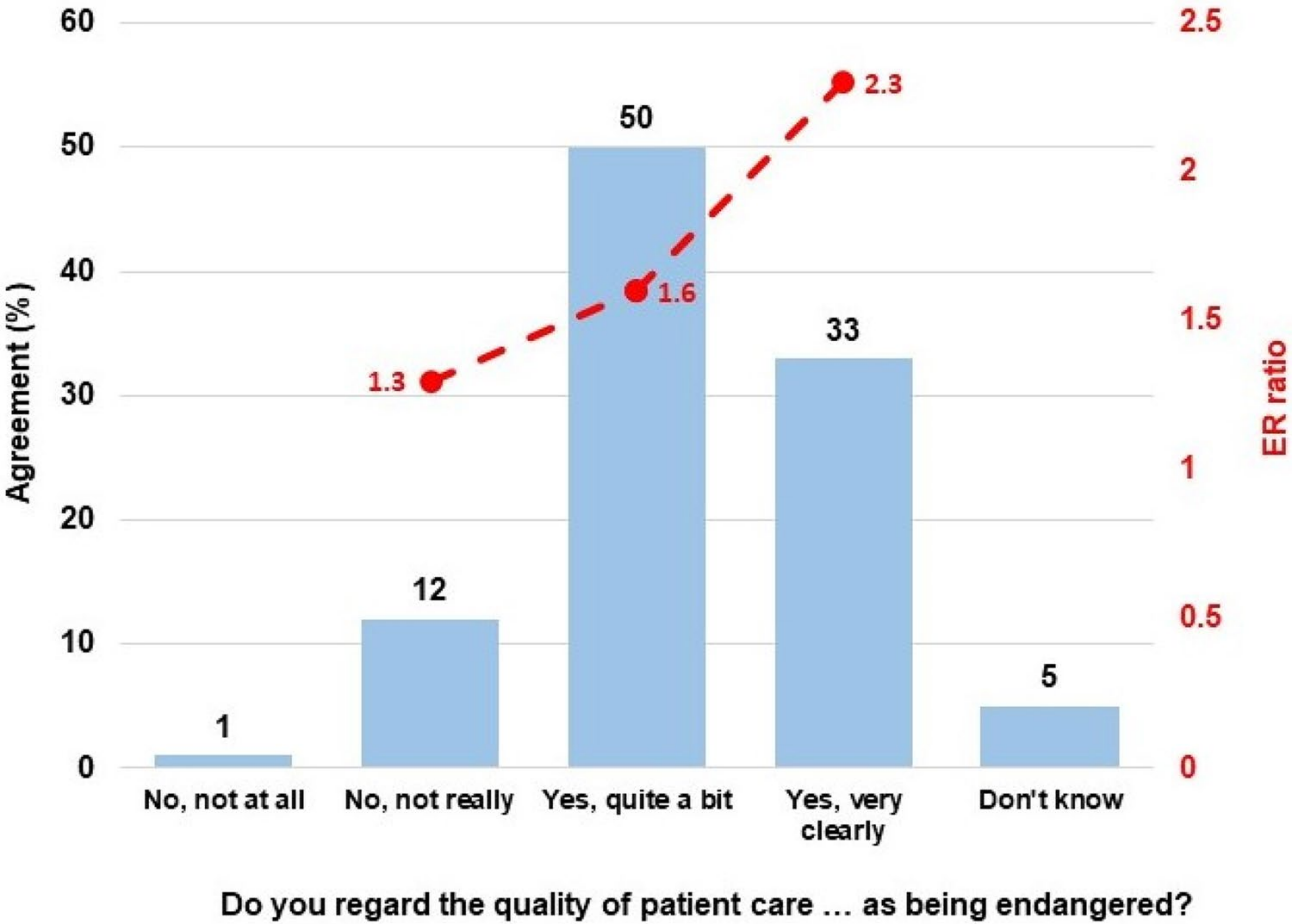
A pessimistic assessment of the quality of care is associated with a high level of psychosocial work pressure. The association between subjectively perceived changes in the quality of care in recent years and the severity of psychosocial work pressure, expressed in terms of the effort–reward (ER) ratio (the options for “No, not at all” (*n* = 3) and “Don’t know” were excluded from the analysis); *P* < 0.001

The participants were asked to assess whether they regarded the quality of patient care as having been endangered by the complex changes in the medical working environment in recent years. One percent (3/391) responded “No, not at all,” 12% (45/391) “No, not really,” 50% (194/391) “Yes, quite a bit,” and 33% (128/391) “Yes, very clearly”; 5% (21/391) answered “Don’t know.” The three most common reasons given for this (only including participants who answered “Yes, …”, with five reasons to choose from, a maximum of three choices, and with a mean of 2.7 responses/participant) were the intensification of work (30%), the increase in nonmedical activities (29%), and insufficient supervision (24%). A more negative assessment of the quality of patient care was associated with a high level of psychosocial work pressure (ANOVA/Tukey, *P* < 0.001, eta-squared 0.11; the groups “No, not at all” (*n* = 3) and “Don’t know” were excluded from the analysis; group differences were each *P* < 0.001, except for “No, not really” vs. “Yes, quite a bit” at *P* = 0.12) (Fig. 1).

The participants were also asked whether, and how often, it happens in everyday working life that economic considerations affect their medical and professional decisions. This was not the case for 10% (38/391), rarely for 34% (133/391), weekly for 36% (142/391), and almost daily for a further 18% (71/391); 2% (7/391) said they did not know. The greater the economic impact on medical decisions was regarded as being, the greater the participants regarded the risk to the quality of patient care as being (chi-squared, *P* < 0.001, Cramér’s *V* 0.2, with the answer option “Don’t know” excluded from the analysis in each case).

Forty-eight percent of the participants (187/391) had actually taken advantage of at least one of the following options due to dissatisfaction with their working conditions (multiple choices possible): 42% (79/187) had already reduced their working hours, 45% (84/187) had changed their job, 5% (9/187) had given up practical medical work, and 8% (15/187) had moved abroad. Asked about average weekly working hours, one participant stated that they were less than 20 h a week, 18% (71/391) 20–40 h, 26% (100/391) 41–50 h, 34% (133/391) 51–60 h, and 22% (86/391) said they were working more than 60 h a week. Depending on the level of care provided by the institution, the hours worked clearly differed to the disadvantage of large institutions (chi-squared, *P* < 0.001, Cramér’s *V* 0.26, “Don’t know” option excluded from the analysis). In university hospitals, for example, 45% of the respondents were working more than 60 h a week, in comparison with 11% in primary-care hospitals.

With regard to job satisfaction (Kruskal–Wallis, *P* = 0.37) and psychosocial work pressure (ANOVA, *P* = 0.05, *α*_6_ = 0.008) there were no statistically significant differences in relation to weekly working hours. Seventy-four percent of the participants (289/391) stated that their overtime hours were not fully remunerated. Asked about the principal mode of compensation, 11% (44/391) stated that they were paid overtime, 45% (178/391) said they received compensation in the form of spare time, 24% (92/391) were both paid and also received compensation in the form of spare time, and 20% (77/391) gave other details. The monthly extra workload for night and/or weekend shifts was zero for 3% (11/391), 1–3 occasions for 8% (33/391), 4–6 for 57% (224/391), 7–9 for 25% (99/391), and more than 10 for 1% (5/391); 5% (19/391) were on permanent shift work.

### Medical specialty training

**Fig. 2:**
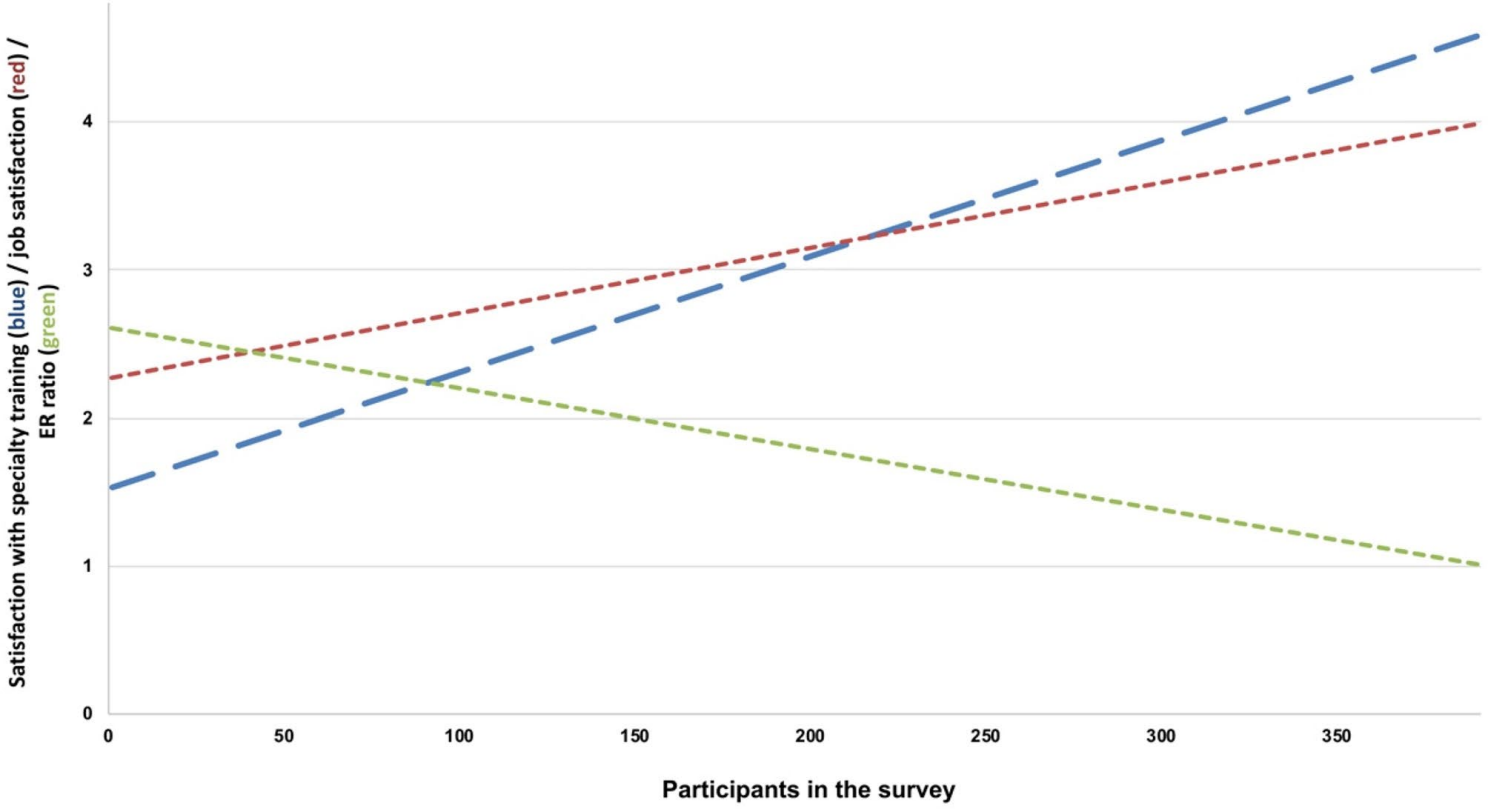
Correlation between satisfaction with medical specialty training and general job satisfaction and psychosocial work pressure. On the horizontal axis, the 391 respondents are shown in ascending order of satisfaction with their medical training (blue line, “very dissatisfied” = 1 and “very satisfied” = 5). A high level of satisfaction with the specialty training correlates positively with general job satisfaction (red balance line, Spearman’s rho 0.52, *P* < 0.001, “very dissatisfied” = 1 and “very satisfied” = 5), and correlates negatively with the extent of psychosocial work pressure (represented as the effort–reward ratio, green balance line, Spearman’s rho − 0.38, *P* < 0.001). *ER* effort–reward ratio

General satisfaction with their specialty training, 4% (16/391) were very dissatisfied, 25% (99/391) rather dissatisfied, 35% (137/391) undecided, 32% (125/391) quite satisfied, and 4% (14/391) very satisfied. A high level of satisfaction with specialty training correlated positively with overall job satisfaction (Spearman’s rho 0.52, *P* < 0.001) and with a lower level of psychosocial work pressure (Spearman’s rho 0.38, *P* < 0.001) (Fig. 2).

The following frequencies were reported in relation to opportunities for specialty training (multiple answers possible; these options do not necessarily mean that the physicians were aiming for specialty training in any specific subspecialty, it simply indicates that training in these clinical fields was available): general gynecology 92% (359/391); obstetrics 90% (351/391); gynecological oncology 68% (264/391); breast diseases 57% (221/391); urogynecology 53% (206/391); prenatal medicine 51% (198/391); and endocrinology/reproductive medicine 21% (81/391). Forty-three percent of the participants (167/391) had received an employment contract for the entire period of their specialty training at the very beginning of residency.

In relation institutional level of care, the most striking difference was between university hospitals (8% yes vs. 92% no) and maximum-care hospitals (61% yes vs. 39% no; chi-squared, *P* < 0.001, Cramér’s V 0.41). At the start of their specialty training, 82% of the participants (321/391) were unable to grasp the structure of their planned specialty training (for example, the sequence of rotations and their approximate time points). Structured resident programs were associated with higher job satisfaction (MWU, *P* < 0.001, *r* 0.21). No statistically significance in relation to psychosocial work pressure was detected (*t* test, *P* = 0.08).

Fifty-six percent of the participants (220/391) considered that they had not learned the specified competencies at the end of the regular specialty training period. When they were asked for the reasons (up to three out of four options, with a mean of 1.8 answers per participant), the following frequencies were noted: 34% (146/426) stated that there were insufficient opportunities for learning diagnoses/interventions, 31% (132/426) that there were too few learning reviews, 19% (80/426) that rotations did not allow the required content to be learned, and 16% (68/426) that there were too few rotations.

Asked about the most beneficial specialty training methods (stating the three most frequent, with up to three choices out of 10 options, with an average of 2.9 responses per participant), the participants stated: 26% supervision by superiors, 18% a structured curriculum, and 15% regular feedback from training personnel. Twenty-six percent of the participants (101/391) stated that the mandatory progress evaluations that ought to take place at least annually (known as *Weiterbildungsgespräche*) had not been realized at all only by 28% (109/391); a further 46% (181/391) stated that they were irregular. The realization did not have any impact on job satisfaction (Kruskal–Wallis, *P* = 0.07). The same also applied to psychosocial work pressure (ANOVA, *P* = 0.09). Eighty-one percent of the interviewees (317/391) did not think that these progress evaluations would improve their specialty training. Twelve percent of the participants (48/391) stated they had completed, or were intending to complete, part of their specialty training on a part-time basis; 35% (136/391) did not know whether they would. Given the availability of the relevant structures, 60% (238/391) would like to do part of their medical specialty training on a part-time basis; 19% (74/391) did not know.

### Surgery and procedures in gynecology and obstetrics

**Fig. 3:**
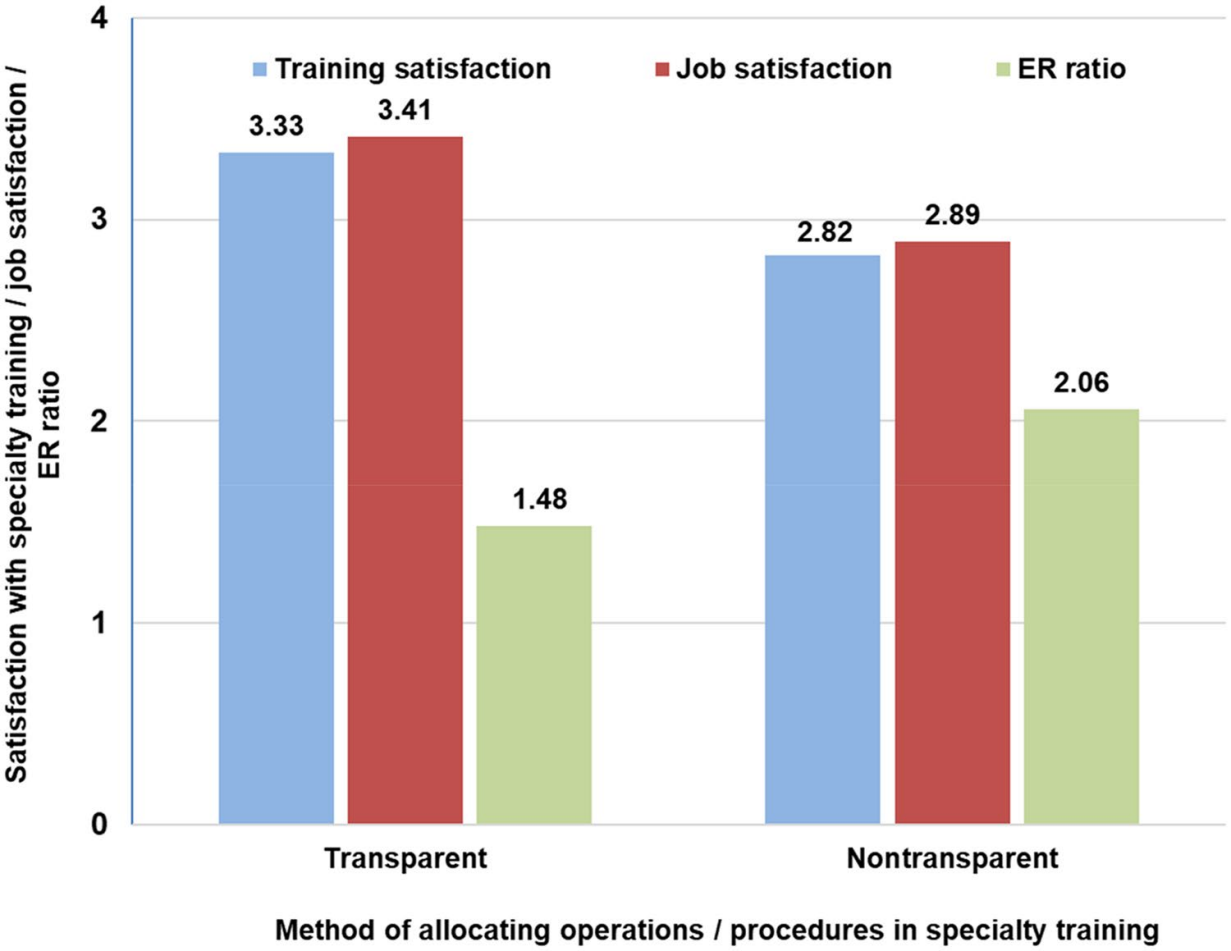
Association between transparent allocation of operations and procedures in medical training with higher levels of satisfaction with the training and general job satisfaction, as well as lower psychosocial work pressure (effort–reward ratio). The diagram shows means for training and general job satisfaction (possible answers: “very dissatisfied” = 1 and “very satisfied” = 5; calculations were based on medians; see text for further explanations), *P* < 0.001 in each case. *ER* effort–reward ratio

In all, 93.6% (366/391) said they had been involved in the operating room during the previous year, and 86% (337/366) had worked in the obstetrics/delivery room. Table 3 provides an overview of the frequency of procedures performed and operations carried out by respondents within the previous year. The participants were asked to assess whether operations/procedures carried out during medical specialty training are distributed transparently and fairly. Fifty-four percent (210/391) answered in the negative, 33% (130/391) answered in the affirmative, and 13% (51/391) abstained. Transparent allocation of procedures was associated with a higher level of satisfaction with the specialty training (MWU, *P* < 0.001, *r* 0.26), greater general job satisfaction (MWU, *P* < 0.001, *r* 0.26), and less psychosocial work pressure (*t* test, *P* < 0.001, 95% CI 0.35–0.81, Cohen’s *d* 0.54, with the option “Don’t know” excluded for each answer) (Fig. 3).

**Table 3:** Frequency of procedures and operations performed by the respondents within the previous year

Frequency of colposcopies carried out during the previous year (*n* = 391)	
None	68%
1–9	21%
10–30	7%
≥ 31	4%
Frequency of minor operations on the external and internal genitalia and chest carried out as an operating surgeon in the previous year (*n* = 366)	
None	4%
1–9	15%
10–30	42%
31–60	27%
≥ 61	14%
Frequency of major vaginal and abdominal procedures carried out as an operating surgeon in the previous year (*n* = 366)	
None	44%
1–5	27%
6–10	14%
11–20	8%
≥ 21	7%
Frequency of vaginal surgery carried out as an operating surgeon in the previous year (*n* = 337)	
None	37%
1–5	39%
6–10	13%
≥ 11	13%
Laparoscopic interventions carried out as an operating surgeon, in total (*n* = 391)	
None	3%
Acting as assistant	23%
1–9	31%
10–30	23%
31–60	13%
≥ 61	7%
Number of births independently attended to during the previous year (*n* = 337)	
None	2%
1–9	2%
10–30	11%
31–60	32%
61–100	34%
> 100	19%
Number of cesarean sections independently performed during the previous year (*n* = 337)	
None	6%
1–9	19%
10–30	46%
31–60	25%
61–100	4%

When asked who was responsible for allocating the surgeries/procedures in practice, 22% (84/391) mentioned the head of department, 39% (152/391) the leading consultant, 27% (107/391) another consultant, 5% the ward doctor, and 8% (30/391) other people. Information about the normal method of allocation was also requested (with a multiple choice among five options and an average of 1.4 options per participant): “allocation according to subjectively estimated surgical needs” (175/512) and “no identifiable pattern” (173/512) were both reported by 34% each, “preferential allocation” by 21% (105/512), “allocation based on target agreements” by 6% (32/512), and “allocation by statistics/counts” by 5% (27/512).

### Work–life balance

Thirty-eight percent of the respondents (150/391) had a child or children at the time of study participation. Forty-seven percent (70/150) had one child, 39% (58/150) had two children, and 15% (22/150) had three or more. The children were under 3 years old in 50% of cases (75/150), aged 3–6 in another 25% (38/150), and over 6 years old in 25% (37/150). Female participants (336/391) were asked about working during pregnancy and about activities they would perform during pregnancy. A distinction was made between the actual and the desired situation (with a multiple choice among nine options; a mean of 4.9 options per participant were selected for “actual” and a mean of 5.6 per participant for “desired”). The most common “actual” options were working on the ward, doing administrative work, and patient admission, each at 19%, and carrying out gynecological examinations of patients at 18%. The four most common “desired” options, each at 17%, were working on the ward and admitting patients, and gynecological examinations and administrative work, each at 16%. Invasive procedures were chosen more frequently as “desired” options—e.g., working in the operating room, at 4% vs. 10% (actual vs. desired), or conducting birth procedures at 5% vs. 8%. Seventy-seven percent of the female participants (258/336) would like to continue working in the operating room during pregnancy if appropriate safety precautions were consistently taken (for example, radiation protection, handling of anesthetic gases).

Fifty-three percent of all survey participants (206/391) reported that working with reduced weekly hours in their own hospital was or would be possible without problems; a further 38% (147/391) stated that it was or would be possible with some restrictions; and another 4% (16/391) stated that it was not or would not be possible. Childcare facilities were not available in house for employees for 50% of the participants (194/391), with limitations for 30% (115/391), and without any restrictions for 12% (46/391); 9% (36/391) said they did not know.

### Compatibility of everyday clinical work with research and scientific work

Fifty-four percent of the participants (212/391) already had their doctoral thesis, and another 33% (128/391) were planning or working on a dissertation. Thirty-two percent (126/391) said they were currently doing scientific work. The proportion of those who were doing scientific research, or intending to do so in the future, was closely related to the type of hospital involved (chi-squared, *P* < 0.001, Cramér’s *V* 0.49). The largest proportion was observed in university hospitals, at 53%, with maximum-care hospitals following, at 32%. The proportion of male doctors doing research or wishing to do so was approximately twice as high as among female doctors (60% to 40%, respectively, among 55 male doctors vs. 28% to 72% among the 336 female doctors; Chi-squared, *P* < 0.001, Cramér’s *V* 0.24).

Working full-time (275 full-time employees vs. 116 part-time employees: 39% vs. 16%, chi-squared, *P* < 0.001, Cramér’s *V* 0.22) and not having any children (241 employees without children vs. 150 employees with children: 42% vs. 17%; chi-squared, *P* < 0.001, Cramér’s *V* 0.25) were also associated with a higher proportion of physicians doing research. Those who were not doing scientific research gave the following three main reasons for this (with a multiple choice possible among six options and a mean of 2.2 answers per participant): 28% “had too little time or thought other things were more important”; 24% thought “there is no time for it alongside hospital work and family”; and 20% stated that “in my hospital there is no opportunity to do so.” Among the researchers (126/391), 5% (6/126) stated that they were very satisfied; no participants said they were only fairly satisfied; 68% (85/126) said they were rather dissatisfied; and 28% (35/126) said they were very dissatisfied with the current or foreseeable conditions for doing their own scientific work. Seventy-eight percent of those who were dissatisfied (94/120) gave reasons for this (with a multiple choice possible among seven options and a mean of 2.8 answers per participant). The three most common reasons given were that the doctors were doing research in their own leisure time, at 30% (77/260); that there was a general lack of guidance and support, at 17% (45/260); and that there was a lack of material and personal support, at 16% (41/260). Finally, 79% (95/120) of the researchers who were dissatisfied with conditions commented on what would make research more attractive for them (with a multiple choice possible among nine options and a mean of 2.6 responses per participant). The three most common aspects mentioned were provision of more time for this during specialty training, at 30% (75/247); provision of additional training courses in scientific skills, at 25% (61/247); and support with the choice of a scientific subject, at 12% (29/247).

## Discussion

The present study provides an overview of stress factors in various important areas in the working life of young doctors in gynecology and obstetrics. The general level of job satisfaction among the physicians surveyed was mainly rated as neutral to positive. This result is familiar from similar surveys and can be interpreted as representing a high level of basic identification with the medical profession, despite the clear criticism expressed at the same time.

### Psychosocial work pressure

All things considered the psychosocial pressure of work was high among our study (ER ratio 1.8) and is comparable with that in recent studies addressing young doctors in other disciplines within Germany (e.g., ER ratios of 1.4 in urology [15], 1.6 in anesthesiology [16], and 1.9 [11] or 1.8 [17] in internal medicine), but is well above the mean values for the working population in Germany (0.6) [18]. The work-expierence i.e. educational level correlated with higher level of psychosocial work pressure. One explanation might be an increase in responsibilities and tasks in the private sphere (for example, due to starting a family or buying real estate) as well as at work (for example, taking a leadership position), and decreasing tolerance with age for states of affairs perceived as unsatisfactory. There was also a pronounced tendency toward overcommitment among the participants that is known to exacerbate existing psychosocial work pressure. In the analysis of the reward scale, job security—in line with the current situation on the labor market—was rated highest (and thus least problematic), in contrast to esteem and promotion opportunities.

It is known that a high level of psychosocial work pressure has far-reaching consequences that are relevant for the health-care system and for patient care in general. It is associated with increased staff turnover (45% of doctors in this survey had already changed jobs due to dissatisfaction) [19], with an increased risk of mental illnesses such as depression and burnout syndrome [13, 20, 21], and with reduced rates of subjective quality of care (> 80% of physicians included in this survey regarded the quality of patient care as being at risk) [22–26].

Physicians’ own state of health has recently come into focus in debates over health-care policy. For example, the President of the World Medical Association issued an urgent warning on the high prevalence of burnout syndrome, pointing out that almost half of the world’s 10 million physicians may be showing symptoms of burnout [27]. The 122nd German Medical Conference, held in Münster in 2019, also discussed the agenda item “When work makes doctors ill.” A recent charter for physicians’ well-being defines factors that should enable physicians to work in a healthy and effective way and argues that it is only in this type of working environment that doctors can sustainably ensure high-quality and safe patient care [28].

### Working time and organization of work

Long working hours are consistent with those of the 2017 Monitor of the German physicians’ association *Marburger Bund,* which also calculated an average working week of 51 h for physicians [5]. The same also applies to the burden of high monthly amounts of shift work, the lack of compensation for extra work required, and work intensity. The results for our cohort are less favorable than those of the *Marburger Bund* study including all groups of physicians. One reason for the intensification of work is the time required for documentation and administrative tasks, in addition to economization and commercialization in the hospital—case numbers increased by 13% between 2007 and 2017, while bed numbers and lengths of stay decreased by 1% and 12%, respectively [29] after the introduction of the DRG system. Twenty-two percent of daily working hours are spent on nonmedical activities, it is comparable with data from internal medicine (30%) [11, 17].

To allow consistent recording of working hours, an automatic electronic procedure needs to be established nationally. Shift work and on-call duties must be adequately remunerated (financially or through other forms of compensation). Optimized general conditions in the health-care sector must reduce workloads (e.g., by delegating nonmedical activities using electronic solutions that actually relieve the burden on physicians and thus reduce documentation requirements); the American College of Physicians (ACP) has formulated detailed recommendations [30] for this purpose to reduce the economic pressure (e.g., by moving away from control mechanisms based on quantity toward a value-based approach to health care).

### Medical specialty training

The most common reason stated for dissatisfaction at work was the poor quality of specialty training. Learning through the personal transfer of knowledge directly at the patient’s bedside has become increasingly difficult in in-patient care in today’s conditions, since staffing does not allow these double structures (with specialty training taking place on the same patient with whom trainees are working). Increasing intensification of work and a lack of (financial and staffing) compensation for the provision of specialty training leave little scope for this. Corresponding findings have also been reported in similar surveys of other specialties and organizations [4, 5, 11, 15–17]. Since transparent allocation in the planning of surgery is associated with greater satisfaction and less psychosocial work pressure, comprehensible, transparent, and fair criteria need to be applied to achieve this.

Specialty training urgently needs to be given greater structure and control. In a systematic description of fundamental conflicts arising in the field of medical schools and specialty training, van den Bussche et al. [31] have called for educationally well-founded specialty training, as is now standard in medical reform study courses and model study programs. In addition, medical faculties and regional medical associations need to work together more closely to ensure a continuum of professional medical development from the first year of study to specialist qualification (and beyond). Education contracts provide physicians with certainty that the employer is intending to lead them (at least) as far as a specialist qualification and also has longer-term plans with them. It would be conceivable to establish an incentive system that would reward the provision of good-quality further specialty training and provide special resources for specialty training. Until that becomes possible, the medical boards of registration must regularly review and firmly limit or revoke authorization for providing specialty training if there are any indications of organizational and structural limitations (in terms of staffing, actual numbers of interventions relative to the number of physicians employed in education, etc.). It should be made possible for physicians to receive medical specialty training with a structured curriculum wherever it is offered. The previous catalog for medical specialty training (2003 version, latest update 2015 [32]) stipulates numbers of interventions to be included in the standard further training period that can only be realistically achieved in extremely few hospitals (above all the 100 major surgical interventions). In the new 2018 version of the catalog for medical specialty training [33], the numbers have been adjusted (in relation to the major surgical interventions, for example, “participation” is now sufficient). It remains to be seen whether these numbers will allow adequate surgical training. In the field of obstetrics, it still appears to be possible to achieve the numbers of interventions required.

The minimally invasive interventions are of great importance in gynecology. The use of laparoscopic techniques is the gold standard for benign gynecological diseases. The training of the corresponding surgical procedures should commence in the specialist training. 366 of the 391 respondents were used in the operating room last year (94%). The majority of participants (57%) performed less than 10 laparoscopies in one year.

In 2016 Gabriel et al. [34] surveyed 109 resident junior doctors on laparoscopic training within specialist training in Germany. 87% of respondents consider learning minimally invasive techniques to be very important. When asked “Are you satisfied with the state of your current basic or advanced endoscopic training?” 62% replied that they were partly or inadequately satisfied. The training of laparoscopic techniques in the period of specialist training does not seem to take place to the extent necessary or expected.

### Job and family

Gynecology is a broad field that has a very high proportion of female physicians and is therefore in a special position. Departments that do not offer part-time working will soon scarcely be able to find sufficient staff. Individual risk assessments are only permitted to restrict the desired areas of employment for pregnant women in the case of unjustifiable hazards. Neither European legal regulations nor the German ones generally exclude surgical work during pregnancy [35].

### Scientific work

The 54% rate of doctoral degrees corresponds approximately to the national average for physicians, at 60% [36]. The fact that only 53% of the questionnaire respondents are also involved in university scientific research, or wish to be in the future, indicates that conditions for scientific work are not optimal. In addition, almost all respondents involved in research (96%) were dissatisfied with the research conditions provided. The major reasons were a lack of protected time and a lack of support, that have been previously described in international studies in other specialties [37, 38].

Intensive and prolonged working days in hospital are obstacles to the establishment of a scientific career—particularly in a period when research is technically and methodologically highly demanding, internationally well networked, and highly competitive. Male gender, full-time work, and not having any children were associated with an increased likelihood of being involved in research work in the present study. These factors are particularly counterproductive in the female-dominated environment of gynecology [39, 40]. Early and generous research funding is needed to give physicians sufficient scope for scientific work (for example, in the form of clinician-scientist programs). The new regulations for specialty training now for the first time allow scientific research to be credited to the specialty training period for up to a total of 12 months [33], and this reduces the disadvantages of long research periods in relation to medical specialty training.

### Limitations

The present study has some limitations that should be borne in mind. The proportion of unvalidated and potentially suggestive questions may have led to a bias in the results. The low participation rate may limit the representativeness of the sample. Potential selection bias may be present, since more dissatisfied physicians may have taken up the invitation to participate in the survey, and the physicians responding to the questionnaire were mainly those who were members of the specialist society. The short version of the questionnaire on the model of occupational gratification crises also tends to overstate the severity of such crises.

## Conclusion

In a health-care system in which almost everything is now being measured and priced, there is one resource that appears to be easy to exploit without any limitations—the professionalism of those who are actively providing patient care. The stress factors in the working life of young physicians are diverse in gynecology and obstetrics and in some cases very pronounced. High-quality medical specialty training and conditions that allow and provide physicians with a healthy and effective working environment are necessary to sustainably maximize the “physician” as a professional resource and to ensure high-quality patient care.
