# Correction to: The work and training situation for young physicians undergoing specialty training in gynecology and obstetrics in Germany: an assessment of the status quo

**DOI:** 10.1007/s00404-021-06142-3

**Published:** 2021-08-02

**Authors:** Johannes Lermann, Julia Knabl, Johannes Neimann, Kevin Schulte, Kim Proske, Sarah Schott, Matthias Raspe

**Affiliations:** 1 Department of Gynecology and Obstetrics, Erlangen University Hospital, Friedrich Alexander University of Erlangen–Nuremberg, Universitätsstrasse 21-23, 91054 Erlangen, Germany; 2 Department of Obstetrics and Perinatal Medicine, Hallerwiese Clinic, Nuremberg, Germany; 3 Specialist in Gynaecology and Obstetrics, 31228 Peine, Germany; 4 Department of Internal Medicine IV/Renal and Hypertensive Diseases, Schleswig-Holstein University Hospital, Kiel Campus, Kiel, Germany; 5 Department of Gynecology and Obstetrics, Heidelberg University Hospital, Heidelberg, Germany; 6 Department of Infectious Diseases and Respiratory Medicine, Charité-Universitätsmedizin Berlin, Corporate Member of Freie Universität Berlin, Humboldt-Universität zu Berlin, and Berlin Institute of Health, Charitéplatz 1, 10117 Berlin, Germany

## Correction to: Archives of Gynecology and Obstetrics (2020) 302:635–647 10.1007/s00404-020-05616-0

The article “The work and training situation for young physicians undergoing specialty training in gynecology and obstetrics in Germany: an assessment of the status quo” written by Johannes Lermann, Julia Knabl, Johannes Neimann, Kevin Schulte, Kim Proske, Sarah Schott and Matthias Raspe was originally published electronically on the publisher’s internet portal on May 26, 2020 without open access. With the author(s)’ decision to opt for Open Choice the copyright of the article changed to © The Author(s) 2020 and the article is forthwith distributed under a Creative Commons Attribution 4.0 International License, which permits use, sharing, adaptation, distribution and reproduction in any medium or format, as long as you give appropriate credit to the original author(s) and the source, provide a link to the Creative Commons licence, and indicate if changes were made. The images or other third-party material in this article are included in the article’s Creative Commons licence, unless indicated otherwise in a credit line to the material. If material is not included in the article’s Creative Commons licence and your intended use is not permitted by statutory regulation or exceeds the permitted use, you will need to obtain permission directly from the copyright holder. To view a copy of this licence, visit http://creativecommons.org/licenses/by/4.0/. Open Access funding enabled and organized by Projekt DEAL.

The original article has been updated.

